# Needs for a Curricular Change in Primary and Secondary Education From the One Health Perspective: A Pilot Study on Pneumonia in Schools

**DOI:** 10.3389/fpubh.2021.654410

**Published:** 2021-11-16

**Authors:** Francisca Marchant, María Pilar Sánchez, Ximena G. Duprat, Alejandro Mena, Marcela Sjöberg-Herrera, Soledad Cabal, Daniela P. Figueroa

**Affiliations:** ^1^Department of Chemical Engineering and Biotechnology, Center for Biotechnology and Bioengineering (CeBiB), University of Chile, Santiago, Chile; ^2^Department of Biology, Faculty of Chemistry and Biology, University of Santiago, Santiago, Chile; ^3^One Health One World Laboratory, Applied Research Center of Chile (CIACHI), Science and Education Foundation, Santiago, Chile; ^4^Faculty of Veterinary Medicine, University Mesoamericana, Puebla, Mexico; ^5^Department of Molecular Genetics and Microbiology, Faculty of Biological Sciences, Pontificia Universidad Católica de Chile, Santiago, Chile; ^6^San José of the Precious Blood High School, Quinta Normal, Santiago, Chile; ^7^Ecophysiological Modelling Laboratory, Liberal Arts Faculty, Adolfo Ibáñez University, Santiago, Chile; ^8^Eco-models & Climate Change Laboratory, Applied Research Center of Chile (CIACHI) Science and Education Foundation, Santiago, Chile

**Keywords:** alternative conceptions, school vulnerability index, pneumonia, public health, One Health education

## Abstract

This is the first pilot study on alternative conceptions and obstacles pertaining to pneumonia in adolescents of different school vulnerability indexes. Countries with low socioeconomic levels are disproportionately affected, with Latin America and the Caribbean (LAC) being the second-most affected area in the world, after sub-Saharan Africa. In spite of this fact, pneumonia is not included as an important component within the contents of the microbiology curriculum unit in the natural science school program. Therefore, we wanted to study how students knew about this topic by putting One Health into action by building and validating qualitative and quantitative questionnaires, put together by different experts in pedagogy, didactics, microbiology, and veterinary to find out what students knew about pneumonia and their misconceptions about it. A total of 148 students (in 8th and 9th grade) participated in this survey. The results reveal that no statistically significant differences between the different scholar grades (*p* = 0.3360 Pearson chi^∧^2) or genders (*p* = 0.8000 Fisher's exact test) presented higher or lower School Vulnerability Index (SVI). Regardless of the social stratum or the level of vulnerability of the students, they have heard about this disease primarily through their family/relatives, maintaining a superficial notion of the disease, learning wrong ideas about microorganisms and treatments that can contribute to the risk to public health.

## Introduction

Pneumonia is a common and potentially serious infection that has a significant prevalence in childhood and causes 15% of all deaths of children under 5 years of age (1). When pneumonia is acquired in a community environment, it is called community-acquired pneumonia (CAP). This disease can be caused by bacteria, such as *Streptococcus pneumoniae, Haemophilus influenzae, Mycoplasma pneumoniae, Chlamydophila pneumoniae*, and viruses including SARS-COV2, Human parainfluenza viruses, and Influenza viruses, among others (2). Countries with a low socioeconomic level are disproportionately affected by CAP, with Latin America and the Caribbean (LAC) being the second most affected area in the world, after sub-Saharan Africa (3). Usual contact with pets is a risk factor for CAP (4) and this is a serious health problem that could be mitigated with adequate access to nutrition, water, energy, clean air, immunization, health, and education services under the One Health approach (5, 6).

In order to design measures to mitigate the impact on health of a disease such as COVID-19, it is important to understand the causal factors, the infectious cycle, and its transmission (7, 8). Education plays a predominant role in understanding these factors, since infectious diseases can be prevented with basic biosecurity measures. Therefore, education must provide scientific literacy to society, fostering critical thinking when facing events such as pandemics, because in the age of the anthropocene, processes like urbanization, globalization, and industrialization have made the world more vulnerable to pandemics than ever before (9). One Health can help provide an effective international “antidote” to such pandemics (10) and serves as an ideal framework for developing problem-focused curricula that promote interdisciplinary teamwork (11).

Undoubtedly, receiving education about infectious diseases from an early age is of great importance, as it helps to improve alertness in children, reducing the risks of contamination due to harmful microorganisms that may get in contact with them and also their animals (pets and cattle) (12). An early introduction to the One Health educational experience to students will allow them to have a more complete and integrative vision on health issues, as it happens in some high school programs in Sweden and the United States (13).

Then, to begin education, it is important to first measure the notions that a person possesses, either by experience or by what they have learned in their schooling years, which are called alternative conceptions (AC). The importance of considering the ideas that students bring to the classroom lies in the need to guide their learning toward the construction of knowledge. This can be accomplished through scientific research work, including creative activities of scientific work, the formulation of hypotheses or the elaboration of experimental designs (14). An obstacle to overcome, for both children and adults, is difficulty in understanding how you get an infectious disease, what causes it to spread, and how it can be prevented (12).

Although the AC reveals the way in which children have represented the natural phenomena with whom they have been involved, it often happens that these conceptions present mistakes. This is a great obstacle and generally involves an incoherence between the interpretations of the world and scientific knowledge. This type of science-related obstacles in AC can be explained by multiple factors; for example, one obstacle can be the culture through which the child was raised as a student (15, 16). For this reason, the purpose of this work is to know the AC on pneumonia (human/animal) in children of different vulnerability indices, in order to expand the knowledge and show to the ministries the importance of educating in One Health from the first years of school.

## Methods

A cross-sectional pilot study was used to evaluate the effects of health education on knowledge about (human/animal) pneumonia in Chilean secondary students, through the comparison of AC. The target population of this study comprised 8th grade (13–16 years) and 9th (13–17 years) grade school students located in Estación Central, La Florida, and Quinta Normal communes in the Metropolitan Region, Chile. Each municipal school was requested to take a survey, and 4 out of 15 responded and authorized taking it (sample of convenience). These communes were selected according to the school vulnerability index (SVI), from high vulnerability (SVI1) to low vulnerability (SVI3) (17).

The survey consisted of 12 questions broken down into two parts: (i) characterize the student's AC on pneumonia (human/animal) as a concept and mental model in adolescents through three open-ended questions, and (ii) measure AC through nine closed questions with four alternatives (Supplementary Figures 1, 2). The survey was given in a biology class for a period of 20–30 min in duration, previously agreed with the teacher/professor of that class. The students were seated in isolation and all doubts were clarified before taking the survey. The survey was building by different experts in pedagogy, didactics, microbiology and veterinary and validated by experts in microbiology and biological science teaching, according to: (a) feasibility (characteristics associated with the time spent completing the survey, the format, the interest, clarity and briefness of the questions, as well as the ease of scoring); (b) reliability (characteristic related to reproducibility); and (c) validity (refer to the ability of a survey to measure what it has been designed).

Once the survey was completed, the data obtained were analyzed externally by an expert in biostatistics. To perform the analysis of the information collected, ATLAS.ti v.7.5.7 software was used to process and analyse the information related to drawings and explanations of the students. Data entry was performed using Microsoft Excel 2010 (Microsoft Office, Redmond, WA, USA) for determining the percentage of correct answers in each of the participants in this study. Stata MP v 13.1 software (STATA Corp LP, USA) was used for statistical analysis. A significance level of α = 0.05, was considered in all statistical tests applied in this study. All the variables of the study were summarized as mean and standard deviation or frequency and percentage.

This research was approved by the Research Ethics Committee of the University of Santiago of Chile. All participants indicated their willingness to participate in this research, with the consent of their parents and the authorization of each school. For this purpose, the names of each student were omitted, in order not to expose the privacy of their participants, which were sequentially labeled from school 1 to 4.

## Results

A total of 15 educational establishments were selected according to SVI, but only 4 (26.7%) of them met the inclusion criteria (informed consent obtained from the participants prior to the survey) to be part of this research. Sixty-nine students belonged to 8th grade and a total of 79 students were in 9th grade. The age range of the 8th grade students fluctuated between 12 and 15 years, while that of the 9th grade students varied between 14 and 17 years of age (Supplementary Table 1). In total, six groups of students were studied: three groups corresponding to 8th and 9th grade, each one classified in SVI1 to SVI3.

According to the different parts of the survey, the answers of varying complexity were obtained, both about the drawings and their respective explanations about pneumonia, as well as the responses selected in the alternative questions. The survey results were classified into categories: qualitative (part I) and quantitative (part II).

In relation to the characterization of AC about pneumonia (part I), within the most prevalent answers, and according to the qualitative analysis, it was determined that for both 8th and 9th grade students, the physiological-anatomical approach was the one that predominated the most in the sample studied. It was characterized by drawings like the Supplementary Table 2A, some of the answers from 8th and 9th grade students in accordance with the SVI related to the organs and structures that make up the respiratory system (nose, pharynx, bronchi, lungs, thorax, and diaphragm, among other parts). In this Table, the PRS code was assigned to indicate “Physiological Respiratory System.” This explanatory model accounts for the macroscopic vision that students have with respect to pneumonia.

It would be expected to find in 8th grade students and in 9th grade students (who were taught microbiology in 7th grade and cell in 8th grade), slightly more complex explanatory models that consider concepts such as prokaryotic cell, bacteria, virus, infection, immune system, contagion, and among others.

**Figure 1:**
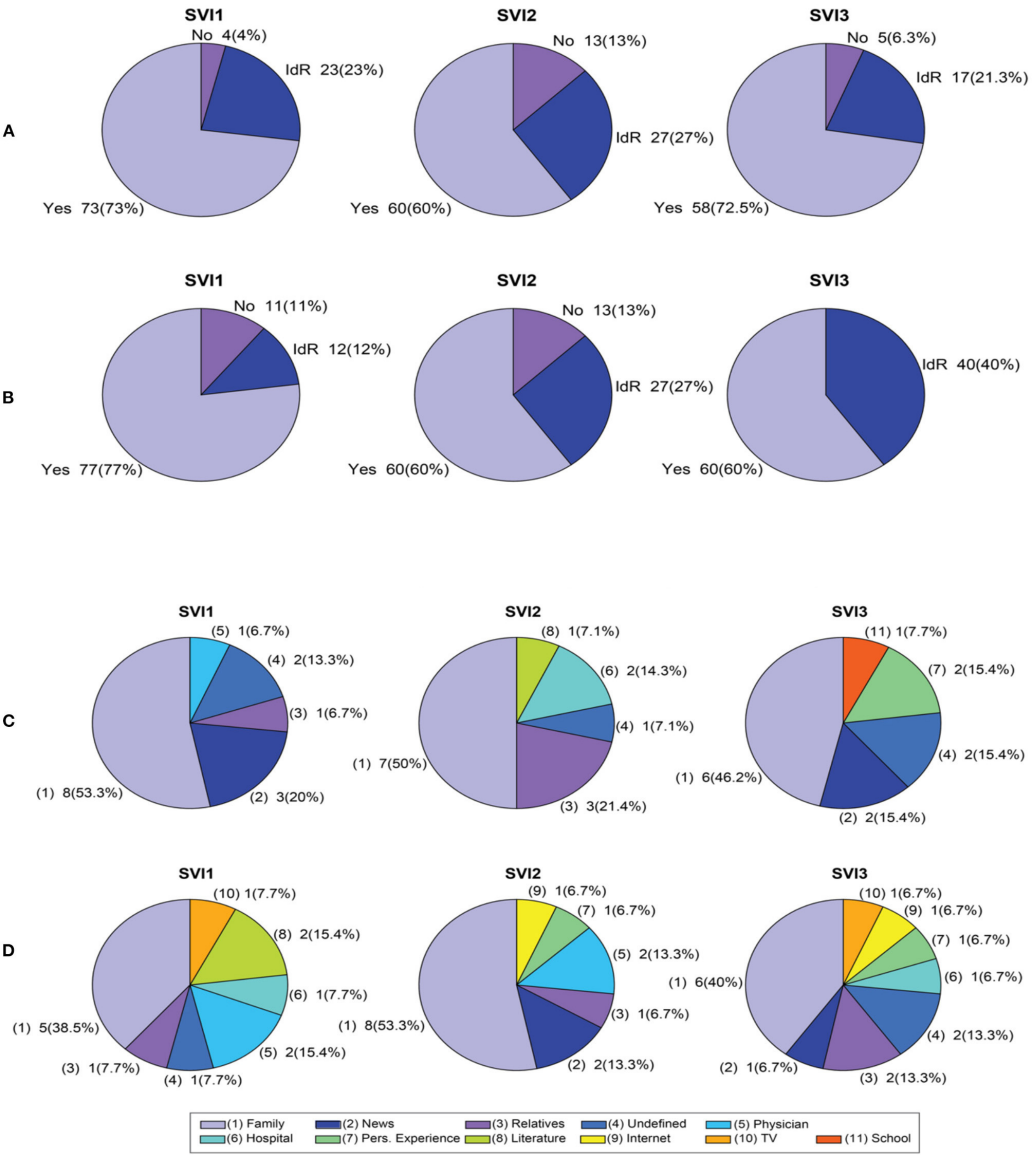
Distribution of responses (in %) according to SVI, Question 2: Have you heard about pneumonia? (Part 1), **(A)** 8th and **(B)** 9th. Frequency distribution of responses by SVI according to source of information Question 2: How did you know? (Part 2), **(C)** 8th and **(D)** 9th. *Zero values were excluded in the construction of the **(C,D)**.

Interestingly, students in both levels (8th and 9th) with the greatest vulnerability (SVI1) have heard more about pneumonia (Figures 1A,B) and found out through a physician from their own experience, that is, because the children became ill with pneumonia. Students in the 8th grade provided a wider variety of responses, while in 9th grade, the AC was clearer that pneumonia is mainly related to the lungs. According to the knowledge about pneumonia (part II), <20% admit not having heard about pneumonia, all of them belonging to high vulnerability schools (SVI1). The most common source of information about pneumonia in both levels (8th and 9th) was the family, and only one student mentioned the school as a source of information (Figures 1C,D, zero values were excluded in the construction of the Figure).

Another AC is related to the belief that the bloodstream is the main transmission mechanism of pneumonia when, in reality, it is saliva droplets contaminated with the pathogen, which can come into contact with the host in multiple ways (SVI1).

Despite the fact that students recognize that the best measure in case of suspected pneumonia is to consult a physician, this option was selected as the second most frequent in all groups. Self-medication with antibiotics dominated as AC in the students from the most vulnerable schools and is accompanied by the idea that getting vaccinated once sick, it could be a measure to consider as possible in case of having the disease. Although most students understood that antibiotics are the adequate pharmacological treatment for pneumonia, most students also answered that the disease was caused by viruses and must be treated with antibiotics, which is contradictory as antibiotics are not used to treat viral diseases.

Additionally, vaccines appear as the second most frequent option for treatment. This is also an obstacle in their explanatory models because vaccines are not exclusive to viruses. In relation to comparison of the number of correct answers in schools according to SVI, when comparing the number of correct answers at the different levels, that there are no statistically significant differences with *p*-values obtained; almost all are greater than α = 0.05 (significance level). The only exception occurred when comparing the percentage of correct answers between the 8th grade schools with SVI2 and SVI3. In global terms, it can be observed that the most vulnerable students answered a lower number of correct answers than the least vulnerable students, both in 8th and 9th grade.

When comparing schools according to their vulnerability index and educational level (Supplementary Table 2B), statistically significant differences were found only for the 8th grade (Kruskal–Wallis test, *p* = 0.0313). Schools with a vulnerability index that gave statistically significant differences were schools with SVI1 and SVI3 and schools with SVI2 and SVI3 (Dunn test), *p*-value 0.0423 and 0.0044, respectively. In the case of 9th grade, no statistically significant differences were found (Kruskal–Wallis, *p* = 0.2078). These results indicate that the different SVI have no relationship on the type of AC that the students in this research may have on pneumonia, and that regardless of the socioeconomic and cultural context in which these contents are taught, students are likely to have similar notions about the disease.

The distribution of the responses of the surveys applied and answered is shown in Supplementary Tables 3–5 (Correct survey choices, total correct answers, and distribution according to SVI and level).

## Discussion

Among the main findings of our research, we detected that the students have the concept associated with pneumonia at the physiological-anatomical level, while we would expect to find that the students consider the explanatory models a little more complex in relation to the causes of the disease. In addition, students belonging to schools with greater vulnerability have less knowledge of the concept of pneumonia and their main source of information is family (relatives). Interestingly, we found that students were unaware that the third cause of death in Chile is pneumonia, which is caused mainly by bacterial agents. This information was less known among students from institutions with a higher degree of vulnerability. On the other hand, regarding the measure in a suspected case of pneumonia, most of the students knew they should go to a physician, but at least half indicated that it could be treated with antibiotics, vaccines, and even a healthy diet. This result may have been affected by the curricular contents of Natural Science of 8th and 9th grade. For 8th grade, the most relevant systems of the human body were taught, including the Respiratory System, a reason that could explain why the 9th grade students responded more accurately.

Also, students thought that viral pneumonia can be treated with antibacterials and that vaccines are only used to treat viruses (18). This AC contains two issues: (i) the vaccine is associated as a treatment and not as a preventative tool, and (ii) pneumonia was caused by a virus, which could explain why students consider the vaccine as a treatment against this type of pathogens.

These misconceptions are the main problem that can contribute to public health risk (19) and increase resistance to antibacterials. In general, we found that students who participated in this study have similar notions about pneumonia regardless of their SVI. Perhaps this is due to the lack of understanding of the real scope of this infectious disease in the community, due to the low coverage and quality of education in Chile (20, 21). Chile performs academically below the average of the OECD countries (22). To improve this perception, science teachers must be able to project contextualized teaching in new social settings, with the aim of re-educating citizens so they are capable of facing the future (23). Considering that Chile has the highest incidence of SARS-CoV-2 worldwide with more than 29,803 cases per million (24), it is important to educate the population about the diseases, learn to recognize its symptoms in time, have access to vaccines and precautions, and avoid infection as much as possible. In the training, identification of principal signs of relative diseases that can be transmitted from animals to humans (zoonoses) need to be considered, so they will be prepared to prevent outbreaks and dissemination, protecting themselves (8).

Therefore, it is necessary to implement the development of a public policy for health education with the One Health work strategy. This requires an interdisciplinary approach (animal, human and environmental professionals) optimizing the use of available resources (25). In this sense, One Health approach programs have been designed and implemented through workshops that use activities based on experience and research to teach concepts of pathogen transmission, disease risk assessment, and mitigation (26).

The term pneumonia could be used with the name human/animal pneumonia for the students (must of them won't know that are the same pathogens), because the etiology and clinical signs are similar, so this could help in the detection of pneumonia not only in humans, also in animals because they can be infected with pneumonia and the students will be prepared to report or make an alert about this disease in the animal field too.

Pneumonia can affect domestic animals that can be in contact with humans, like dogs, cats, and cattle. Etiological agents like *Bordetella bronchiseptica, Rhodococcus equi*, or *Capnocytophaga canimorsus* can be found in the oral cavity of these animals and be transmitted to humans through bites or direct contact with fluids (27). This is important under the One Health approach because this type of study, and the integration of disease information to the signatures can be implemented with other infections, like vector-borne diseases, zoonoses, etc. So if the students are trained in their schools, they will be able to detect on time, prevent and report diseases in humans and animals. This could be key in their own protection and prevention of future pandemics.

For example, OH Sweden has developed an educational strategy program to promote understanding the interaction between pathogens, hosts, and the environment in a didactic way, through the interaction of students and researchers (13). The OH Training and Leadership program improves household and personal hygiene practices and animal housing in low-income, high-risk communities in South Africa (28). Rwanda has developed educational tools on One Health at the government level in its environmental, livestock and health plans; encouraging the resolution of problems related to infectious diseases in all professions (25). The implementation of an educational system with the One Health concept allows for an early diagnosis and timely treatment of pneumonia, and a reduction of hospital expenses (~$6,000 US) (29). Like other emerging diseases, One Health concept can also allow preventative measures to mitigate the presence of pneumonia through exposure to urban air pollutants (30).

Chilean public policy for education must implement the One Health concept and it should be located at the level of: Ministry of Education, all providers of public health services (Ministry of Health), financial agents (Ministry of Economy) and the scientific community.

Our study has some limitations: This research only focuses on one discipline, so transdisciplinary perspectives can generate hypotheses that a discipline perspective may miss. Second, we only collected data on knowledge of pneumonia without gender differentiation. This data is important to consider, as women have been reported to be more concerned with maintaining hygiene compared to men (31). Despite these limitations, this study demonstrates the importance of infectious disease education and provides a reference to promote preventive behaviors among Latin American students. This study provides valuable information on the issues that must be prioritized and improved to prevent infectious diseases. We propose the establishment of strategic actions integrated by a network of various agents with the objective of improving education in Chile under the One Health concept.

## Data Availability Statement

The raw data supporting the conclusions of this article will be made available by the authors, without undue reservation.

## Ethics Statement

The studies involving human participants were reviewed and approved by Research Ethics Committee of the University of Santiago of Chile. Written informed consent to participate in this study was provided by the participants' legal guardian/next of kin.

## Author Contributions

SC and DF conceived and designed the project. SC performed experiments. SC, MS, and FM analyzed the data. MS, DF, and FM interpreted the data. DF, XD, FM, and MS discussed data and wrote the manuscript. DF, MS-H, MS, AM, and XD reviewed and edited the manuscript. All authors read and approved the final manuscript.

## Funding

MS was supported by the research grant CONICYT/FONDECYT/REGULAR No. 1171004.

## Conflict of Interest

The authors declare that the research was conducted in the absence of any commercial or financial relationships that could be construed as a potential conflict of interest. The handling editor is currently organizing a Research Topic with one of the authors DF.

## Publisher's Note

All claims expressed in this article are solely those of the authors and do not necessarily represent those of their affiliated organizations, or those of the publisher, the editors and the reviewers. Any product that may be evaluated in this article, or claim that may be made by its manufacturer, is not guaranteed or endorsed by the publisher.
