# Supplementary material for: Needs for a Curricular Change in Primary and Secondary Education From the One Health Perspective: A Pilot Study on Pneumonia in Schools
Source: Front Public Health. 2021 Nov 16;9:654410. doi: 10.3389/fpubh.2021.654410 (PMC8634956; doi:10.3389/fpubh.2021.654410)
Supplement: Supplementary file 1 [file Data_Sheet_1.pdf]

Supplementary Material

Supplementary Figure 1.

**SURVEY FOR STUDENTS**

Part 1 – Alternative Conceptions

Name: ______________________________ Course: _____ Age: _____ years Gender: M___ F___ Other___
Educational establishment: ____

(1) What image comes to your head when you hear the word "Pneumonia"? Then draw what you imagine and then explain your drawing.

Explain what you understand by "Pneumonia"

____

____

(2) Have you ever heard of "Pneumonia"? Mark with an X. (If your answer is “No”, go to the next question)..
Yes ____
Not ____
I don’t remember ____

(3) If you selected "Yes", how did you find out?

____

____

____

Supplementary Figure 2.

SURVEY FOR STUDENTS

Part 2 – Alternative Conceptions

Name: ______________________________ Course: _____ Age: _____ years Gender: M___ F___ Other___
Educational establishment: ____

(1) From the following diseases, which one produces the most deaths of people in Chile?
a. Parasites
b. HIV AIDS
c. Amyotrophic Lateral Sclerosis
d. Respiratory diseases

(2) Pneumonia is a disease caused mainly by:
a. Virus
b. Fungus
c. Bacterium
d. Parasite

(3) The organ of the body directly affected by pneumonia is:
a. Liver
b. Lung
c. Heart
d. Stomach

(4) At what age is it easier to get Pneumonia in Chile?
a. Newborn
b. From 1 to 3 years
c. 15 to 45 years
d. From 65 years or more

(5) How is Pneumonia spread?
a. By blood route
b. By drops of saliva
c. For sexual intercourse
d. From eating feces

(6) The main symptoms of Pneumonia are:
a. Seizures, vomiting, and dizziness
b. Blurred vision, tiredness, and very thirsty
c. Fever, cough, shortness of breath
d. Left arm pain and rapid heartbeat

(7) What should you do when suspected to be sick with Pneumonia?
a. Eat healthier
b. Get vaccinated to cure it
c. Take antibiotics for a week
d. Going to the doctor for tests

(8) What tests should be done to diagnose pneumonia?
a. Endoscopy of the esophagus
b. Lung X-ray
c. Heart electrogram
d. Blood lipid profile

(9) What is the most common treatment for Pneumonia?
a. Vaccine
b. Surgery
c. Vitamin C
d. Antibiotics

frontiers

Supplementary Table 1.

Age distribution of the respondents according to level of education and gender.

Educational level	Women n=66	Men n=79	Other gender n=3	Total N=148	p-value*
**Age (years)**					
Mean	13.71	14.12	14	13.94	
St. Dev.	0.69	9.91	1	0.84	
Minimum value	13	13	13	13	
Maxim value	15	17	15	17	
**8th grade**	**n=3**	**n=3**	**n=2**	**n=69**	**0.8000**
**Age (years)**					
Mean	13.13	13.58	13.5	13.38	
St. Dev.	0.34	0.81	0.71	0.67	
Minimum value	13	13	13	13	
Maxim value	14	16	14	16	
**9th grade**	**n=35**	**n=43**	**n=1**	**n=79**	
**Age (years)**					
Mean	14.23	14.58	15	14.43	
St. Dev.	0.49	0.73	-	0.65	
Minimum value	13	14	15	13	
Maxim value	15	17	15	17	

** Test exact Fisher, significance level α=0.05*

Supplementary Table 2.

A. Explanatory models on Pneumonia, of the 8th grade and 9th grade students, according to SVI

Sample	**Focus**	**Category**	**Coding**	**Code**	**Student explanation** [a]
8_SVI3	Physiological	Anatomical	Respiratory system	PSR	"That the lungs turn black" (I3_C1_E21)
8_SVI2	Physiological	Anatomical	Respiratory system	PSR	"What I understand by pneumonia is a disease (contagious) which are not treated can be fatal" (I2_C3_E3)
8_SVI1	Physiological	Anatomical	Respiratory system	PSR	"Something to the lungs I think" (I1_C4_E12)
9_SVI3	Physiological	Anatomical	Respiratory system	PSR	"It is a disease of the respiratory system and lungs" (I3_C2_E20)
9_SVI2	Physiological	Anatomical	Respiratory system	PSR	"It is a disease to the lungs" (I2_C3_E26)
9_SVI2	Physiological	Anatomical	Respiratory system	PSR	"It is a disease in which your lungs fill with water" (I1_C4_E3)

[a]*In parentheses, the identification assigned by the researcher for each student, according to SVI(I), School (C) and Enumeration (E).*

B. Comparisons of correct answers by SVI and educational level

Educational Level/SVI	**Correct Answers**	**Kruskal Wallis test, p-value**	**Comparison**	**Dunn Test, p-value**
8th grade				
SVI1	21	**0,0313***	SVI1 vs SVI2	0,2125
SVI2	30		SVI1 vs SVI3	**0,0423***
SVI3	18		SVI2 vs SVI3	**0,0044***
9th grade				
SVI1	17	0,2078	SVI1 vs SVI2	**Does not apply**
SVI2	34		SVI1 vs SVI3	
SVI3	28		SVI2 vs SVI3	

*Significance level α=0.05. *Significative p-values*

Supplementary Table 3.
Correct survey choices, (part 2).

Part 2 _Answer	Text	Correct option
P2_1	Of the following diseases, which is the one that produces the most deaths of people in Chile?	4
P2_2	Pneumonia is a disease caused mainly by:	3
P2_3	The organ of the body directly affected by pneumonia is:	2
P2_4	At what age is it easier to get Pneumonia in Chile?	1
P2_5	How is Pneumonia spread?	2
P2_6	The main symptoms of Pneumonia are:	3
P2_7	What should you do when you suspect that you may be sick with Pneumonia?	4
P2_8	What tests should be done to diagnose pneumonia?	2
P2_9	What is the most common treatment for Pneumonia?	4

Supplementary Table 4.
Correct answer distribution.

Part 2 _Answer	Text	n	%
P2_1	Of the following diseases, which is the one that produces the most deaths of people in Chile?	59	39,86
P2_2	Pneumonia is a disease caused mainly by:	38	25,68
P2_3	The organ of the body directly affected by pneumonia is:	122	82,43
P2_4	At what age is it easier to get Pneumonia in Chile?	24	16,22
P2_5	How is Pneumonia spread?	51	34,46
P2_6	The main symptoms of Pneumonia are:	111	75
P2_7	What should you do when you suspect that you may be sick with Pneumonia?	122	82,43
P2_8	What tests should be done to diagnose pneumonia?	109	73,65
P2_9	What is the most common treatment for Pneumonia?	74	50

Supplementary Table 5.

Correct answers distribution according to SVI and level.

Answer	1		2		3		4		5		6		7		8		9	
	n	%	n	%	n	%	n	%	n	%	n	%	n	%	n	%	n	%
**SVI_1**																		
8th	6	40	5	41,7	14	46,7	4	40	7	50	13	48,1	18	54,5	14	50	12	54,5
9th	9	60	7	58,3	16	53,3	6	60	7	50	14	51,9	15	45,5	14	50	10	45,5
**Total**	**15**	**100**	**12**	**100**	**30**	**100**	**10**	**100**	**14**	**100**	**27**	**100**	**33**	**100**	**28**	**100**	**22**	**100**
**SVI_2**																		
8th	7	26,9	8	38,1	26	47,3	2	20	10	43,5	26	51	25	46,3	23	47,9	15	42,9
9th	19	73,1	13	61,9	29	52,7	8	80	13	56,5	25	49	29	53,7	25	52,1	20	57,1
**Total**	**26**	**100**	**21**	**100**	**55**	**100**	**10**	**100**	**23**	**100**	**51**	**100**	**54**	**100**	**48**	**100**	**35**	**100**
**SVI_3**																		
8th	4	22,2	1	20	12	32,4	1	25	4	28,6	10	30,3	12	34,3	8	25	6	35,3
9th	14	77,8	4	80	25	67,6	3	75	10	71,4	23	69,7	23	65,7	24	75	11	64,7
**Total**	**18**	**100**	**5**	**100**	**37**	**100**	**4**	**100**	**14**	**100**	**33**	**100**	**35**	**100**	**32**	**100**	**17**	**100**
